# Mangrove Phenology From Scale, Data and Species Perspectives

**DOI:** 10.1002/ece3.72788

**Published:** 2026-01-04

**Authors:** Yuhang Wang, Qi Liu, Yaojun Zhu, Wanyu Wen, Si Yang, Minghao Gong

**Affiliations:** ^1^ Wetland Research Center, Institute of Ecological Conservation and Restoration Chinese Academy of Forestry Beijing China; ^2^ Beijing Key Laboratory of Wetland Services and Restoration Chinese Academy of Forestry Beijing China; ^3^ Zhanjiang National Research Station for Mangrove Wetland Ecosystem State Forestry and Grassland Administration Zhanjiang China; ^4^ Department of Geography Lingnan Normal University Zhanjiang China

**Keywords:** land surface phenology, Landsat, mangroves, Sentinel‐2, subtropical, time series analysis

## Abstract

Land surface phenology derived from satellite observations provides an effective means of characterizing vegetation growth dynamics. Mangroves, although ecologically critical, remain understudied in phenology research due to geographic variation, inconsistent dataset selection, and limited understanding of species‐specific patterns. In this study, we investigated mangrove phenology in the northern subtropical mangrove distribution area in Zhanjiang, China, from the perspectives of spatial scale, dataset resolution, and species differences. Using harmonic analysis to reconstruct the Enhanced Vegetation Index (EVI) time series, we assessed phenological trajectories at both regional and plot levels and compared Landsat 8 with Sentinel‐2 observations. Results show that satellite‐derived mangrove phenology exhibits a clear annual unimodal pattern across scales, with growth beginning in February, peaking in July, and continuing through December. Sentinel‐2 outperformed Landsat 8 in capturing phenological signals, reflecting its superior spatial and temporal resolution. Pronounced interspecific differences were also detected: *Aegiceras corniculatum* and *Bruguiera gymnorhiza* exhibited continuous growth from January to December, whereas *Avicennia marina* and *Rhizophora stylosa* showed growth from March to December. Ground‐based litterfall data revealed strong seasonality, with peaks in July–August. Except for *A. corniculatum*, litterfall exhibited a significant positive correlation with satellite‐derived EVI growth patterns (*p* < 0.05), supporting the feasibility of species‐level phenology detection from space. Given the increasing pressures on mangrove ecosystems from climate change and human activity, these findings highlight the importance and practicality of long‐term phenology monitoring using multi‐resolution satellite observations.

## Graphical abstract

This study investigates mangrove phenology in the northern subtropical zone of Zhanjiang, China, using satellite‐derived Enhanced Vegetation Index (EVI) time series. Our results reveal clear annual phenological patterns with significant variation across species and datasets, highlighting the superior sensitivity of Sentinel‐2 over Landsat 8. These findings underscore the feasibility of satellite‐based long‐term monitoring of mangrove phenology at multiple spatial scales.

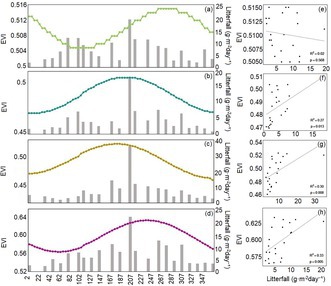

## 1 Introduction

Vegetation phenology, defined as the annual cycle timing of vegetation growth such as leaf unfolding, leaf greenness, flowering, leaf senescence, and litterfall, is crucial for understanding the impacts of climate change on ecosystems, thus attracting increased attention in recent times (Piao et al. 2019). Vegetation phenological dynamics can be examined across multiple spatial scales, from plot‐based studies to regional and global assessments, utilizing extensive phenology data sources including field observations, proximal remote sensing data (e.g., PhenoCams), and satellite observations (Richardson et al. 2018; Schwartz 2003; Xiang et al. 2020). While field‐based observations provide detailed ecological information, they are labor‐intensive and spatially limited. Proximal sensing captures high‐frequency canopy dynamics but remains restricted to small areas. Satellite remote sensing addresses these limitations by offering extensive spatial coverage with frequent acquisitions, facilitating timely monitoring of vegetation dynamics (Hanes 2013; Zeng et al. 2020). Termed land surface phenology (LSP), satellite‐detected vegetation phenology has been widely employed in investigating phenological patterns across diverse ecosystems (Caparros‐Santiago et al. 2021; De Beurs and Henebry 2005; Piao et al. 2006).

Mangrove ecosystems, located in the intertidal zones of tropical and subtropical coastlines worldwide, are recognized for their exceptional carbon sequestration capacity, coastal protection functions, and provision of diverse ecosystem services (Murray 2012; Simard et al. 2018; Wang et al. 2021). Despite their ecological significance, mangroves face increasing threats from habitat loss, coastal development, pollution, and climate change (Fu et al. 2023; van Bijsterveldt et al. 2023; Van der Stocken 2022). Understanding mangrove phenology is critical for advancing ecological research, biodiversity conservation, climate adaptation strategies, and sustainable coastal resource management, thereby supporting the long‐term resilience of both mangrove ecosystems and the human communities that rely on them (Upadhyay and Mishra 2010).

Early studies of mangrove phenology primarily relied on field‐based observations of leaf emergence, abscission, and morphological traits (Steinke and Rajh 1995). More recently, satellite remote sensing has become a key tool in mangrove phenology monitoring. For example, Pastor‐Guzman et al. (2018) used MODIS‐derived spectral indices to track mangrove phenology in the Yucatan Peninsula, while Songsom et al. (2019) compared mangrove phenology to nearby forests using EVI time series in Southern Thailand. Younes et al. (2020); Younes et al. (2021) applied Landsat and Sentinel‐2 imagery with statistical models to identify mangrove phenological patterns across Australia, focusing on variations in the start of season (SOS), peak of season, and phenological curve shape and amplitude. Similarly, Celis‐Hernandez et al. (2022) assessed the effects of pollution on mangrove phenology in the Gulf of Mexico using vegetation indices from Sentinel‐2. Although these studies have advanced our understanding of mangrove phenology, they also highlight considerable variability in key phenological metrics such as the start of season (SOS), end of season (EOS), length of growing season (LOG), and peak growing season (PGS), depending on geographic location, dataset choice, and mangrove species. This variation underscores the need for more targeted studies in underrepresented regions, particularly northern subtropical mangroves. Despite their ecological importance, phenological patterns in these regions remain poorly characterized. Addressing this gap is essential to improving the generalizability and ecological interpretation of satellite‐derived phenology in mangrove ecosystems.

Accurately retrieving mangrove phenology from satellite observations requires careful selection of appropriate datasets, as the spatial, spectral, and temporal characteristics of the sensor directly determine how well key phenological transitions can be detected. Although MODIS has been widely used for phenology studies due to its high temporal resolution and long historical record, its coarse spatial resolution (250–500 m) limits its ability to capture fine‐scale heterogeneity in mangrove ecosystems, particularly in fragmented or narrow coastal habitats (Lu and Wang 2021). In contrast, Landsat (30 m) and Sentinel‐2 (10–20 m) mitigate these limitations, enabling species‐level or stand‐level phenology detection. Recent work has shown that while MODIS, Landsat, and Sentinel‐2 may capture broadly similar seasonal trends, substantial discrepancies arise in derived phenological metrics due to differences in spatial resolution, revisit frequency, and sensor‐specific spectral response functions (Younes et al. 2021). Notably, Sentinel‐2's narrow spectral bands and higher revisit frequency (5 days) compared with Landsat's 16‐day cycle enhance its sensitivity to subtle changes in canopy greenness and leaf developmental stages.

It is important to emphasize that satellite datasets do not modify the intrinsic phenology of mangrove ecosystems, but rather determine how effectively key phenological parameters can be captured and interpreted. Mangroves, with their species‐specific canopy structures, evergreen phenology, and occurrence in narrow intertidal environments, are particularly sensitive to sensor resolution and mixed‐pixel effects. Therefore, systematic evaluation of how different remote sensing configurations influence phenological detection is essential—not only for methodological accuracy, but also for improving ecological interpretation of phenological processes across spatial scales and species.

Beyond data selection, the analytical framework used to reconstruct and interpret time‐series vegetation indices is equally critical for reliable phenological estimation. A wide range of methods—including Gaussian filtering, Savitzky–Golay smoothing, and Harmonic Analysis of Time Series (HANTS) (Chen et al. 2004; Roerink et al. 2000)—have been applied to reduce noise and generate continuous seasonal curves from satellite observations. More advanced approaches such as Continuous Change Detection and Classification (CCDC) (Zhu and Woodcock 2013) and Breaks for Additive Seasonal and Trend (BFAST) (Verbesselt et al. 2010) further decompose time‐series signals into trend, seasonal, and residual components, enabling detection of subtle phenological variations or long‐term shifts. These methods are particularly valuable for mangrove ecosystems, where frequent cloud cover, tidal inundation, and atmospheric variability often obscure true canopy dynamics. Integrating robust time‐series reconstruction algorithms with high‐resolution satellite data can significantly improve the accuracy and ecological relevance of mangrove phenology retrieval.

Although recent studies have begun exploring species‐specific mangrove phenology using remote sensing (e.g., Li et al. 2019; Small and Sousa 2019; Zhao et al. 2024), significant gaps remain. Most existing work has focused on broad regional assessments or relied on coarse‐resolution imagery insufficient for distinguishing phenological differences among co‐occurring mangrove species. Moreover, phenological dynamics in subtropical mangrove ecosystems remain largely understudied, despite field evidence demonstrating strong interspecific variation in flowering, fruiting, leaf turnover, and productivity. Whether such species‐level phenological differences can be consistently detected from space, and how they may interact with spatial scale or dataset characteristics, remains unresolved.

To address these gaps, this study examines satellite‐derived mangrove phenology from the perspectives of spatial scale, dataset resolution, and species variation. We focus on the northern subtropical mangrove distribution zone in Zhanjiang, China, and utilize both regional and plot‐level analyses to explore how phenological patterns vary across spatial scales. Two commonly used satellite datasets—Landsat 8 and Sentinel‐2—are compared to evaluate how differences in spatial and temporal resolution affect the characterization of mangrove phenology. Moreover, we assess species‐specific phenology across four dominant mangrove species using harmonic analysis of Enhanced Vegetation Index (EVI) time series, and validate these patterns with ground‐based litterfall observations.

Specifically, we aim to address the following research questions:

1. Do satellite‐derived mangrove phenology patterns exhibit consistency across different scales?
2. Do different satellite‐derived mangrove phenology datasets exhibit similar patterns?
3. Do satellite‐derived phenology patterns for different mangrove species display similarities?

By answering these questions, this study advances understanding of how mangrove phenology manifests across space, sensor configuration, and species identity, thereby contributing to more robust trait‐based monitoring and supporting improved mangrove conservation and ecosystem management in coastal regions.

## 2 Methods

### 2.1 Study Area

**FIGURE 1:**
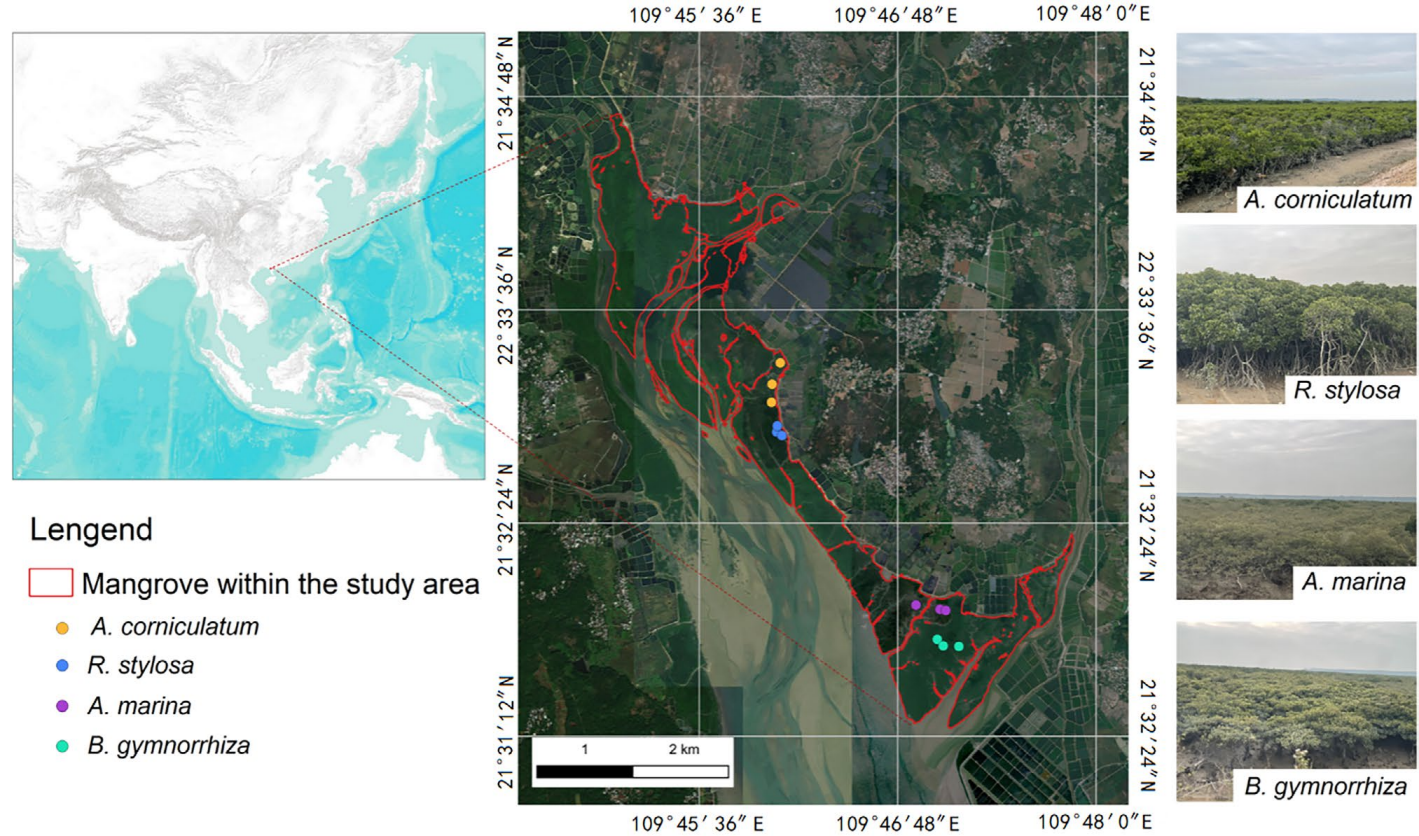
Study area and locations of field plots for the four dominant mangrove species: *A. corniculatum*, *R. stylosa*, *A. marina* , and *B. gymnorhiza* . Photos of the four species are displayed in the corners. All photos were taken by the authors (Yuhang Wang) in the Gaoqiao Mangrove Reserve during field campaigns in 2023. Images used with permission from the photographers.

Mangroves in the northern subtropical regions are primarily located in China, the United States, and Japan. Although Chinese mangroves occupy less than 0.3% of the global mangrove area, they host one‐third of the world's mangrove species diversity. For this study, we selected the mangroves in Zhanjiang, China, due to their extensive distribution and rich species diversity. The study area, Gaoqiao Mangrove Reserve, is situated in the core region of the Zhanjiang Mangrove National Nature Reserve (21°31′‐21°35′ N, 109°44′‐109°48′ E), China (Figure 1). This region experiences a tropical monsoon climate on its northern edge. It is regulated by a maritime climate throughout the year, resulting in mild winters and cool summers. The average annual temperature ranges from 22.7°C to 23.5°C, increasing from north to south, with a difference of 1.5°C between the northern and southern areas. The average annual rainfall is between 1395.5 and 1723.1 mm, and the average annual sunshine hours range from 1714.8 to 2038.2 h. The rainy season lasts from April to September, with August receiving the most rainfall, while the period from October to March is relatively dry. The region is frequently affected by low pressure systems, tropical storms, and typhoons. According to meteorological statistics, from 1960 to 2018, Zhanjiang experienced 52 typhoons, averaging 0.9 per year. The primary mangrove species in the study area are *Aegiceras corniculatum*, *Avicennia marina* , *Bruguiera gymnorhiza* , and *Rhizophora stylosa*.

### 2.2 Data

#### 2.2.1 Field Data

Four dominant mangrove species in the Gaoqiao Mangrove Reserve—*Aegiceras corniculatum*, *Avicennia marina* , *Bruguiera gymnorhiza* , and *Rhizophora stylosa*—were selected as focal species for this study due to their wide distribution (each occupying an area > 0.1 km^2^) and healthy growth condition within the reserve. For each species, three permanent plots (30 × 30 m) were established, with a minimum spacing of 30 m between plots to ensure spatial independence (Figure 1). The precise geographical coordinates of each plot were recorded using a Garmin GPSMAP 66 s handheld GPS unit, which provides sub‐meter accuracy under open canopy conditions (Table S1).

To verify the representativeness and long‐term stability of vegetation within the selected plots, a time‐series analysis of high‐resolution Google Earth imagery from 2014 to 2023 was conducted (Figure S1). Field monitoring of litterfall dynamics was then performed in all plots, ensuring consistent coverage across the four dominant species and capturing interspecific variation in litter production patterns.

Litterfall monitoring was conducted from January 2023 to January 2024 to provide phenological reference data for each mangrove species. Within the distribution of the four selected mangrove communities, four litterfall traps were installed at approximately 1 m above ground to minimize tidal interference in each mangrove community. Traps were placed in locations less likely to be affected by extreme high tides, though we acknowledge that occasional water‐level rise may cause partial loss or drifting of litter, especially in low‐lying areas. This limitation is addressed in the discussion section.

Litterfall was collected at biweekly intervals, sorted into major components (leaves, flowers, pro, branches, and miscellaneous materials), and oven‐dried at 65°C to a constant weight before measurement. The litterfall mass was standardized to grams per square meter per day (g·m^−2^ day^−1^) to derive seasonal accumulation rates and interspecific variation in litterfall production. These temporal patterns were then used as indirect validation of satellite‐derived vegetation phenology metrics, particularly in determining the timing of peak canopy productivity (Pastor‐Guzman et al. 2018). While litterfall data do not provide exact start or end dates of phenophases, their temporal dynamics serve as an important ecological proxy to support and cross‐validate remote sensing‐based phenological interpretations.

#### 2.2.2 Remote Sensing Data

We used time‐series satellite imagery from the Landsat 8 Operational Land Imager (OLI) and Sentinel‐2A/B MultiSpectral Instrument (MSI) to monitor mangrove phenology dynamics. These sensors provide medium‐to‐high spatial resolution optical observations and have been widely adopted in vegetation phenology research. Previous studies have demonstrated that phenological metrics derived from Landsat and Sentinel‐2 exhibit strong consistency in mangrove ecosystems, whereas significant discrepancies exist when compared to MODIS‐derived metrics due to differences in spatial resolution and temporal frequency (Younes et al. 2021).

Landsat 8 OLI imagery offers a 30 m spatial resolution for visible to near‐infrared (VNIR) bands, with a nominal 16‐day revisit cycle. We acquired Level‐2 surface reflectance data (Collection 2 Tier 1) from the Google Earth Engine (GEE) platform. These products are preprocessed by the U.S. Geological Survey (USGS) for radiometric and geometric corrections, orthorectification, and atmospheric correction using the LaSRC algorithm.

Sentinel‐2A and ‐2B MSI sensors operate in a constellation that achieves a 5‐day revisit interval at the equator. The MSI provides imagery at 10 m resolution for visible, NIR, and SWIR bands, and 20 m resolution for the red‐edge bands, which are particularly sensitive to vegetation chlorophyll content. Level‐2A surface reflectance data were obtained from GEE, atmospherically corrected using the Sen2Cor algorithm and distributed by the European Space Agency (ESA) under the Copernicus Open Access License.

All imagery was spatially co‐registered and reprojected to the CGCS2000 coordinate system (EPSG: 4550) to ensure spatial consistency. Cloud and cloud‐shadow pixels were identified and masked using the QA bands provided in each dataset, and time‐series gaps were further reduced by integrating both Landsat 8 and Sentinel‐2 observations.

To characterize vegetation dynamics, we calculated the Enhanced Vegetation Index (EVI) for each valid mangrove pixel in every image within the study period. EVI was selected over the other indices (e.g., NDVI) due to its reduced sensitivity to canopy background noise and superior performance in high‐biomass ecosystems such as dense mangrove forests. The EVI was computed using the following standard formulation:

$$\mathrm{EVI}=2.5\times \frac{\left(\rho_{\mathrm{NIR}}-\rho_{\mathrm{Red}}\right)}{\left(\rho_{\mathrm{NIR}}+6\rho_{\mathrm{Red}}-7.5\rho_{\text{Blue}}+1\right)}$$

where *ρ*_NIR_, *ρ*_RED_, and *ρ*_Blue_ represent surface reflectance in the near‐infrared, red, and blue spectral bands, respectively.

The Landsat 8 and Sentinel‐2 datasets used in this study are publicly available through GEE. Landsat data were provided by the USGS, and Sentinel‐2 data were distributed by ESA. All data processing and analysis were conducted under the Google Earth Engine Terms of Service.

### 2.3 Data Analysis

#### 2.3.1 Overview

**FIGURE 2:**
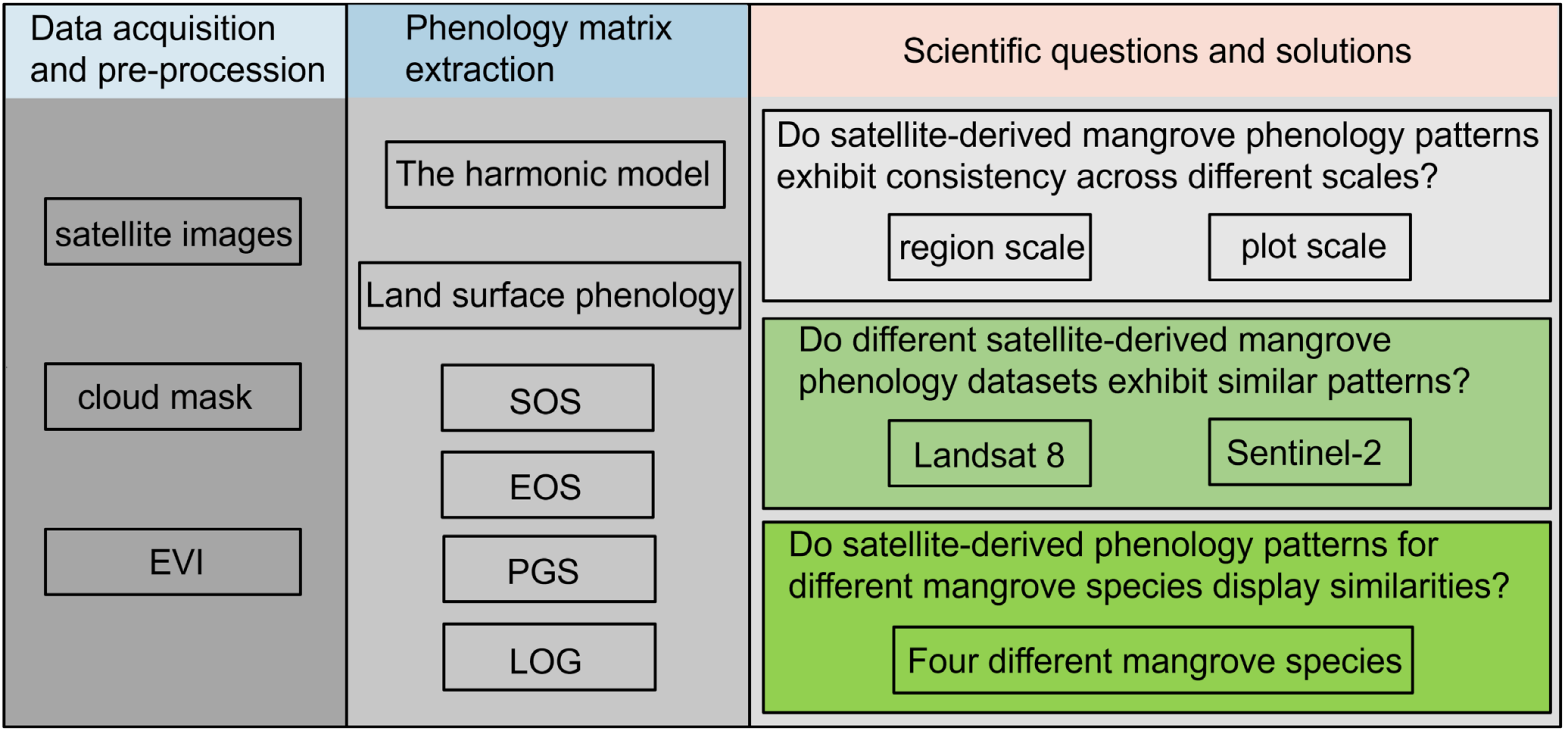
Conceptual diagram of the hypotheses tested.

In this study, we used all available Landsat 8 and Sentinel‐2 imagery at two spatial scales (i.e., region and plot) as shown in Figure 1 to address the three research questions outlined in Figure 2. For each question, we examined key characteristics of mangrove land surface phenology, including the number of peaks and troughs, the median start of the growing season (SOS), median end of the growing season (EOS), median length of the growing season (LOG), median timing of the peak growing season (PGS), and the overall shape of the phenology curve. Each question was analyzed independently, with no interaction tested among spatial scales, datasets, or species. Brief descriptions of these analytical procedures are provided in Sections 2.3.4.

#### 2.3.2 EVI Time Series Reconstruction

Because the EVI values derived from satellite imagery are inherently discontinuous and often contaminated by noise arising from aerosols, cloud cover, and variations in solar–sensor geometry, the original time series required reconstruction prior to phenological analysis. To mitigate these issues, we applied a curve‐fitting approach to generate continuous and smoothed EVI trajectories.

Several curve fitting and smoothing algorithms have been employed in previous mangrove phenology studies, including the Double Logistic and Discrete Fourier Transform (Pastor‐Guzman et al. 2018), Generalized Additive Models (Younes et al. 2020), and Harmonic Analysis of Time Series (HANTS) (Celis‐Hernandez et al. 2022; Li et al. 2019; Valderrama‐Landeros et al. 2021). In this study, we selected the HANTS algorithm due to its superior ability to handle irregularly spaced satellite observations, effectively filter out noisy and cloudy data, and produce stable reconstructions even in high‐humidity coastal regions. Previous research has shown that HANTS performs more robustly than alternative smoothing algorithms such as Iterative Interpolation for Data Reconstruction, Savitzky–Golay, Asymmetric Gaussian, and the Double Logistic functions, particularly under the heterogeneous canopy and illumination conditions typical of mangrove forest (Wu et al. 2021). Harmonic analysis represents periodic temporal signals as a combination of sine and cosine components (Zhou et al. 2015). The HANTS algorithm iteratively fits a least‐squares harmonic curve to the time series, removing outliers that deviate from the expected harmonic pattern (Roerink et al. 2000). During each iteration, data points below the fitted curve are assigned progressively lower weights, allowing the model to converge toward a smoothed, seasonally representative signal (Julien and Sobrino 2019). The general form of the harmonic model is expressed as:

$$y\left(t\right)=a_{0}+\sum_{k=1}^{n}\left[a_{k}\cos\left(\mathrm{kwt}\right)+b_{k}\sin\left(\mathrm{kwt}\right)\right]$$

where *y*(*t*) is the fitted EVI value at time 𝑡, is the mean term, 𝑎_𝑘_ and 𝑏_𝑘_ are the harmonic coefficients, 𝑛 is the number of harmonic components, and 𝜔 represents the fundamental frequency corresponding to one annual cycle.

The HANTS algorithm was implemented in GEE using a modified version of the publicly available community code (https://developers.google.com/earth‐engine/tutorials/community/time‐series‐modeling). The script was adapted to process EVI time series derived from both Landsat 8 and Sentinel‐2 datasets. For each plot coordinate, the algorithm output included both the fitted (harmonic) and observed EVI values, enabling direct evaluation of reconstruction accuracy.

#### 2.3.3 Mangrove Phenology Metrics Extraction

Key phenological parameters—including the start of the growing season (SOS), end of the growing season (EOS), length of the growing season (LOG), and peak of growing season timing (PGS)—were derived from the smoothed EVI trajectories reconstructed using the Harmonic Analysis of Time Series (HANTS) method.

**FIGURE 3:**
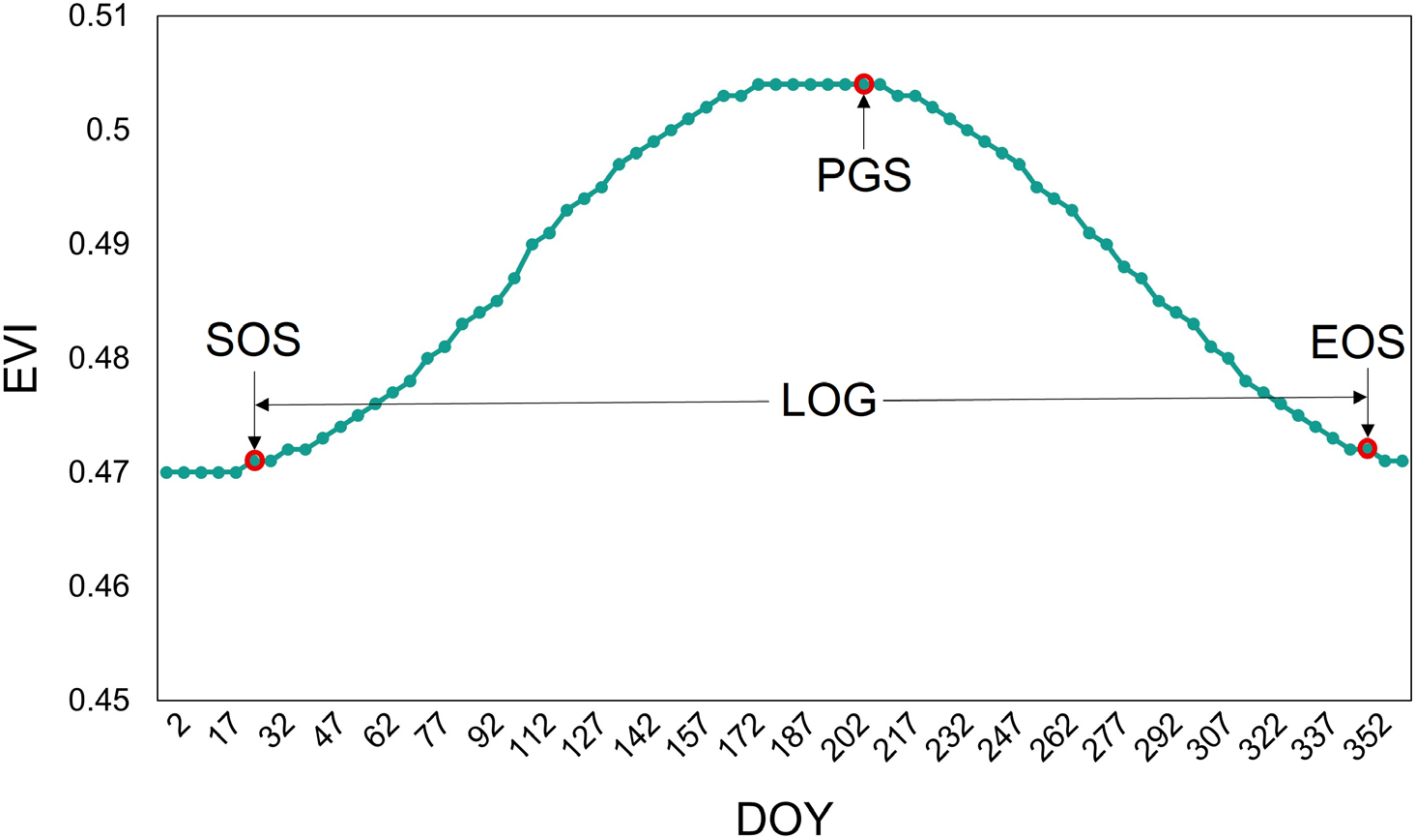
Schematic illustration of mangrove phenological parameter extraction based on the fitted EVIratio time series. SOS is identified as the first day when the rate of EVI change shifts from negative or near‐zero to positive, indicating the onset of canopy greening. EOS corresponds to the final transition from a positive to a negative rate of change within the annual cycle, marking the initiation of leaf senescence. LOG is calculated as the period between SOS and EOS, while PGS is defined as the day of year when the fitted EVI curve reaches its maximum value.

Among commonly used approaches for extracting phenological metrics, two principal strategies are the threshold‐based method and the rate‐of‐change (inflection point) method. To reduce the subjectivity and potential bias introduced by selecting fixed EVI thresholds, this study employed the inflection point approach, in which phenophase transition dates are identified from the first derivative of the fitted EVI time series (Figure 3). Specifically, SOS was defined as the first day of year (DOY) when the rate of EVI change shifts from negative or near‐zero values to positive, marking the onset of canopy greening and the resumption of active photosynthesis. In contrast, EOS was defined as the final DOY within the annual cycle when the rate of EVI change transitions from positive to negative, indicating the initiation of leaf senescence or canopy decline. The LOG was computed as the duration (in days) between EOS and SOS, while the PGS corresponded to the DOY when the fitted EVI curve reached its maximum value, representing the period of peak canopy productivity.

To quantify the rates of EVI change, we calculated the EVI change ratio for each observation using the following expression:

$${\mathrm{EVI}}_{\text{ratio}}\left(t\right)=\frac{{\mathrm{EVI}}_{t+1}-{\mathrm{EVI}}_{t}}{{\mathrm{EVI}}_{t}}$$

where EVI_*t*_ and EVI_*t+*1_ represent consecutive values in the EVI time series, and EVI_ratio_ *(t)* denotes the relative rate of change at time *t*. Positive EVI_ratio_ values indicate canopy development (growing), while negative values correspond to canopy decline (senescence).

For each year within the study period, these phenological metrics (SOS, EOS, PGS, and LOG) were extracted and statistically summarized (mean, median, and standard deviation). The shape of the harmonic EVI curve, including the number and position of peaks and troughs, was further examined to identify intra‐annual fluctuations possibly linked to flowering or defoliation events.

All data processing, curve differentiation, and visualization were performed in R (R 2014) using the packages ‘dplyr’ (Yarberry 2021) for data manipulation and ‘ggplot2’ (Wickham et al. 2016) for graphical visualization.

#### 2.3.4 Satellite‐Derived Mangrove Phenology Analysis at Multiple Scales and Species

To examine the consistency and variability of satellite‐derived mangrove phenology across different spatial scales, data sources, and species, an integrated analysis was conducted using both Landsat 8 and Sentinel‐2 imagery.

##### 2.3.4.1 Scale Definition and Data Selection

Two spatial scales were defined in this study to characterize mangrove phenological patterns at different levels of spatial detail.

- Regional scale: This scale covers an area of several hundred hectares and represents the overall mangrove extent within the Gaoqiao Reserve. It is primarily used to capture large‐scale spatial variability and regional phenological gradients across the entire mangrove ecosystem.
- Plot scale: This scale corresponds to localized mangrove patches and reflects fine‐scale ecological conditions. In this study, the plot scale specifically refers to the established field plots, each measuring 30 × 30 m (900 m^2^). These plots provide high‐resolution information for validating and interpreting regional phenological patterns.

##### 2.3.4.2 Phenology Pattern Comparison Across Scales

Using all available Landsat 8 OLI surface reflectance images from 2014 to 2023, we calculated annual mean Enhanced Vegetation Index (EVI) trajectories for both regional and plot scales. The HANTS algorithm was then applied to reconstruct continuous phenological curves, from which key phenological metrics—SOS, EOS, and PGS were extracted. To characterize long‐term patterns, median values of each phenological metric were calculated across the 10‐year observation period. Temporal consistency between scales was assessed through correlation and phase‐shift analyses, enabling evaluation of whether regional‐scale phenology accurately represents localized canopy dynamics.

##### 2.3.4.3 Comparison of Different Satellite Datasets at Plot Scale

To assess the influence of sensor spatial resolution and temporal frequency on phenology detection, we compared Landsat 8 (30 m resolution, 16‐day revisit) and Sentinel‐2A/B (10 m resolution, 5‐day revisit) EVI time series over the plot scale for the period 2019–2023, when both datasets were simultaneously available. Because of the difference in native resolution, no spatial resampling was performed; instead, each dataset was analyzed at its intrinsic scale. While this approach preserves the spectral integrity of each sensor, it also implies that Sentinel‐2 imagery captures finer‐scale canopy heterogeneity that may not be fully represented in Landsat‐derived phenology. Therefore, comparisons between the two datasets were interpreted qualitatively, focusing on the consistency of seasonal timing rather than absolute EVI magnitude.

##### 2.3.4.4 Species‐Specific Phenology Analysis

To investigate interspecific variability, we extracted species‐specific EVI time series using Sentinel‐2 imagery from 2019 to 2023 within plots dominated by each of the four mangrove species—*A. corniculatum*, *A. marina* , *B. gymnorhiza* , and *R. stylosa*. For each species, the mean EVI trajectory was reconstructed using the HANTS algorithm, and phenological parameters (SOS, EOS, PGS, and LOS) were extracted following the same procedure described in Section 2.3.2.

Given that the regional and plot scales encompass mixed stands rather than pure species canopies, we acknowledge a degree of spectral mixing in the derived signals. Nonetheless, the use of species‐specific plot masks derived from field GPS coordinates substantially reduced potential confusion between species, allowing for the identification of relative differences in phenological timing among the dominant mangrove taxa.

## 3 Results

### 3.1 Mangrove Phenology at Different Scales

**FIGURE 4:**
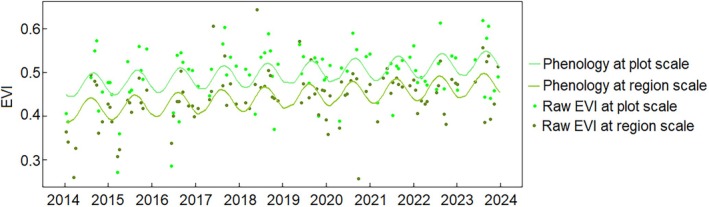
Land surface phenology of mangroves at plot and region scale within Gaoqiao Mangrove Reserve, Zhanjiang, China. Mean values are shown in all panels.

**FIGURE 5:**
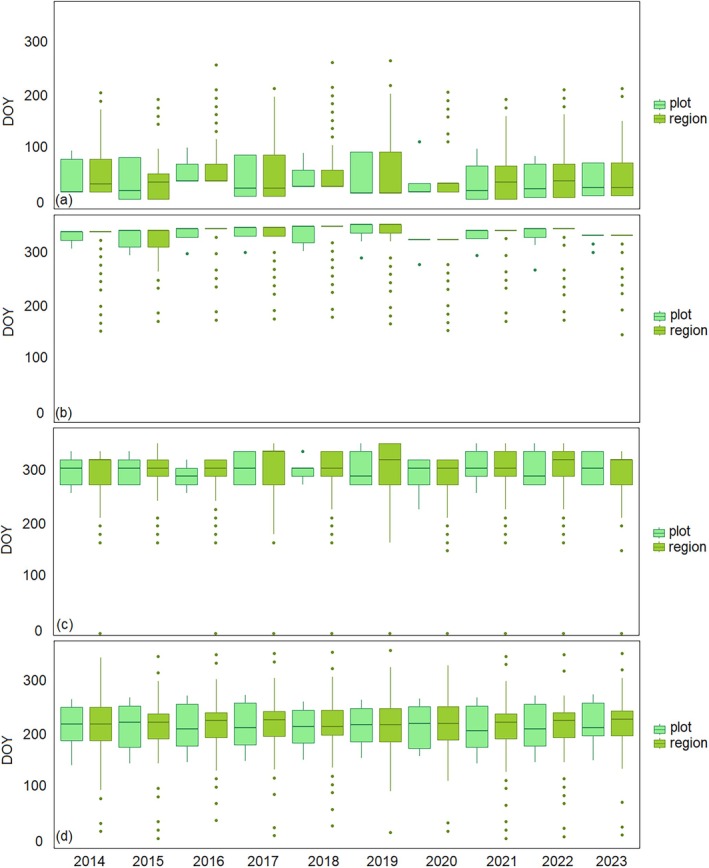
Box plot showing the phenology metrics as determined by the land surface phenology derived from Landsat 8 for mangrove forest within Gaoqiao mangrove reserve from 2014 to 2023 at plot and region scale. SOS (a), EOS (b), LOG (c), and PGS (d).

The harmonic model fitted to the EVI time series successfully captured the primary seasonal dynamics, indicating the periodic behavior of mangroves. Both region and plot scales show distinct mangrove phenology seasonal patterns with a universal curve each year from 2014 to 2023 (Figure 4). However, EVI and fitted EVI values derived from the plot scale were generally higher than those from the region scale. Throughout the study period (2014–2023), the SOS, EOS, and PGS remained relatively stable, showing no clear variation both for the plot and region scales. The phenology metrics SOS, EOS, and PGS for the plot scale show a bit earlier than the region scale (Figure 5).

According to Figure 4 and Table S2, from 2014 to 2024, SOS occurred between January and February (i.e., DOY 14–36 at the plot scale, DOY 12–36 at the region scale) and the EOS occurred in December (DOY 335–364). The PGS identified between late July and mid‐August (DOY 209–225) at the plot scale and in August (DOY 217–231) at the region scale.

### 3.2 Comparison of Satellite‐Derived Mangrove Phenology

**FIGURE 6:**
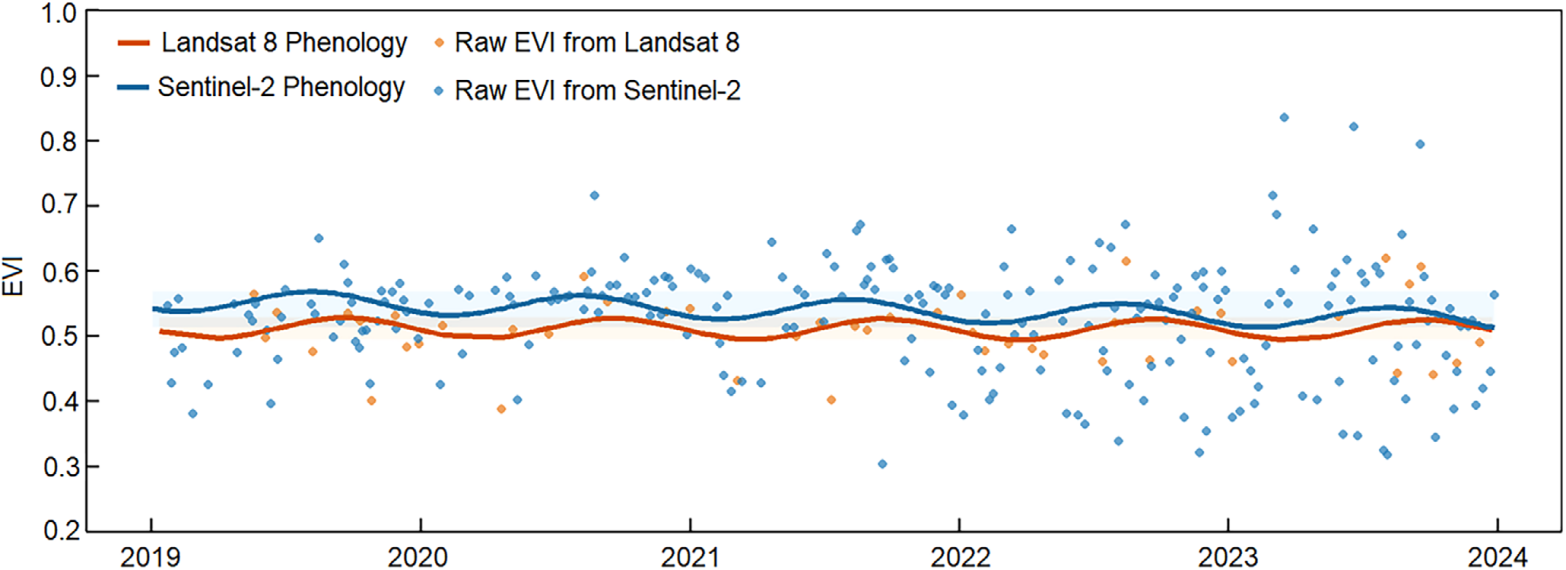
Land surface phenology of mangroves at plot scale within Gaoqiao Mangrove Reserve, Zhanjiang, China. Mean values are shown in all panels.

Land surface phenology derived from Landsat 8 and Sentinel‐2 imagery at the plot scale for the four mangrove species (12 plots) is shown in Figure 6. Due to the higher revisit frequency of Sentinel‐2, 361 usable images were available between 2019 and 2023, compared with only 85 for Landsat 8 over the same period. Both datasets captured the seasonal cycles of mangrove phenology through the harmonic model, with comparable curve shapes (Figure 6). Sentinel‐2 results exhibited greater short‐term variability, while Landsat 8 curves appeared as smoother seasonal cycles.

**FIGURE 7:**
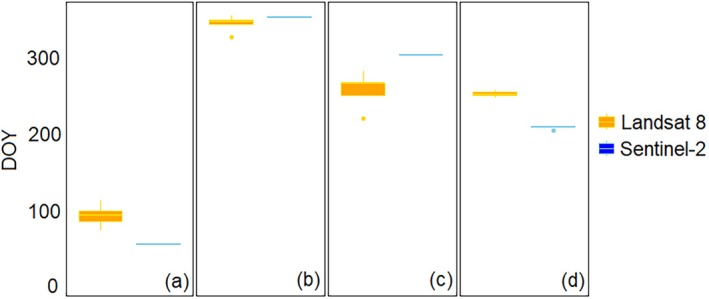
Box plot showing the Peak of Growing Season for mangrove forest at plot scale in Gaoqiao mangrove reserve between 2019 and 2023, as determined by the land surface phenology derived from Landsat 8 and Sentinel‐2. SOS (a), EOS (b), LOG (c), and PGS (d).

According to Figure 7 and Table S3, from 2019 to 2023, Landsat 8‐derived SOS and EOS were detected in March (DOY 71–111) and December (DOY 335–364), respectively, with the PGS occurring in September (DOY 252–263). The LOG ranged from 224 to 288 days. Sentinel‐2 results indicated earlier SOS (February, DOY 52–53) and similar EOS (December, DOY 362–363) with the PGS observed in late July to early August (DOY 207–213). The LOG was approximately 310 days for Sentinel‐2 and 272 days for Landsat 8.

To quantitatively assess the consistency between the two sensors, we compared the fitted EVI time series using Pearson correlation and error metrics. The Landsat 8 and Sentinel‐2 phenology curves showed a moderate positive correlation (*r* = 0.504, *p* = 0.066). The overall discrepancies were small, as reflected by an RMSE of 0.0316 and an MAE of 0.0288 (Figure S2).

### 3.3 Comparison of Species‐Specific Mangrove Phenology Derived From Satellite

**FIGURE 8:**
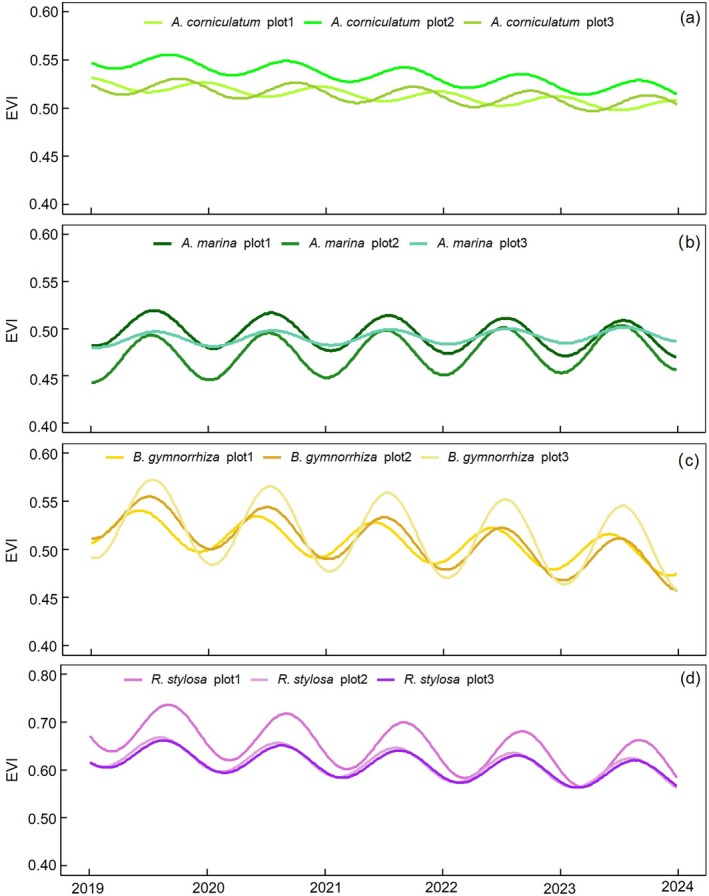
Land surface phenology of four mangrove species at plot scale. *A. corniculatum* (a), *A. marina* (b), *B. gymnorhiza* (c), and *R. stylosa* (d) respectively. Mean values are shown in all panels.

Figure 8 illustrates the temporal variation in the EVI for each species across three different plots from the beginning of 2019 to 2023 based on Sentinel‐2 observations. All four mangrove species exhibited distinct seasonal patterns, though the timing and amplitude of phenological events varied (Figure 8).

**FIGURE 9:**
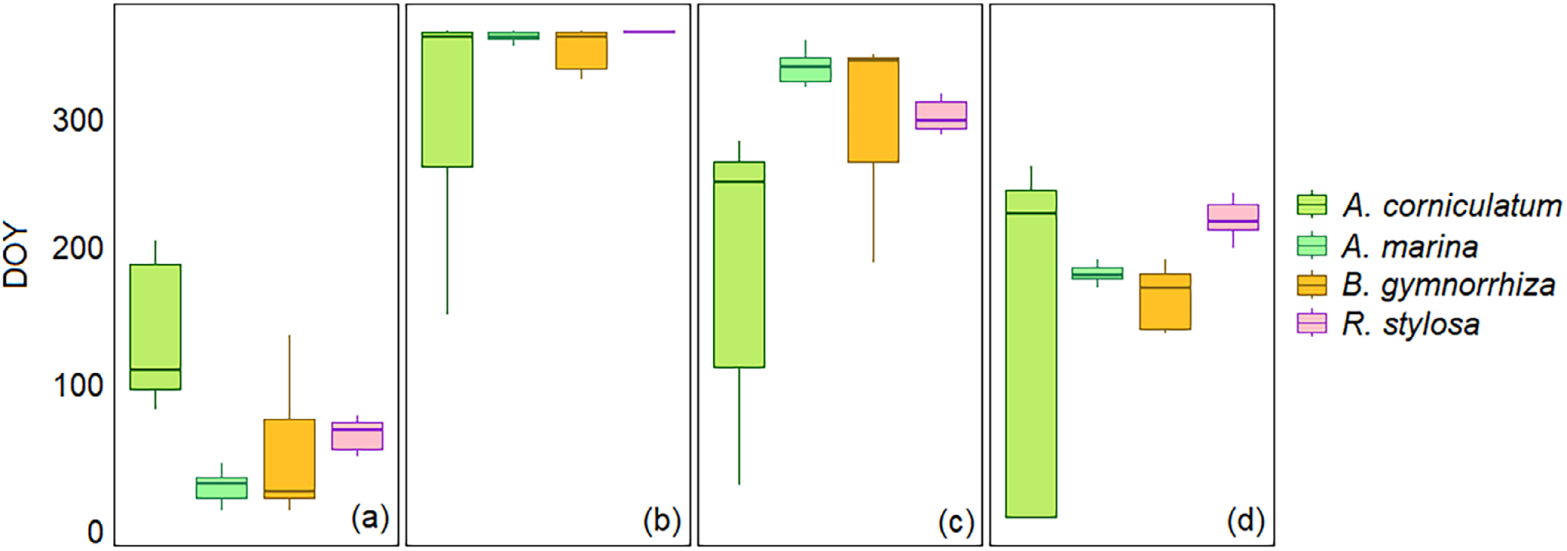
Box plot showing the Peak of Growing Season for four mangrove species at plot scale within Gaoqiao mangrove reserve between 2019 and 2023, as determined by the land surface phenology derived from Sentinel‐2. SOS (a), EOS (b), LOG (c), and (d) PGS.

According to Table 1 and Figure 9, *A. corniculatum* showed a growing season extending from DOY 27 to 358 (January–December), with peak growth typically between August and September (DOY 218–262). *A. marina* began growth around DOY 67 (March) and ended near DOY 362 (December), with peak activity between June and July (DOY 172–193). *B. gymnorrhiza* displayed an extended growing season (DOY 22–358) with peaks from May to July (DOY 138–193). *R. stylosa* started growth later (DOY 112 and 358, March–December) and peaked between July and August (DOY 202–243).

**TABLE 1:** Compilation of median SOS, EOS, LOG and PGS during the study period for each question.

Question	Study site	Data source	Spatial resolution (m)	Period	SOS	EOS	LOG	PGS
Q1: Do satellite‐derived mangrove phenology patterns exhibit consistency across different scales?	Plot	Landsat 8	30	2014–2023	31	348	305	216
	Region				31	353	305	225
Q2: Do different satellite‐derived mangrove phenology datasets exhibit similar patterns?	Plot	Landsat 8	30	2019–2023	92	356	272	257
		Sentinel‐2	10		52	362	310	212
Q3: Do satellite‐derived phenology patterns for different mangrove species display similarities?	*A. corniculatum* plots	Sentinel‐2	10	2019–2023	27	358	336	182
	*A. marina* plots				67	362	296	222
	*B. gymnorrhiza* plots				22	358	341	172
	*R. stylosa* plots				112	358	251	227

*Note:* The median SOS, EOS, LOG and PGS were calculated for each site during the study period.

**FIGURE 10:**
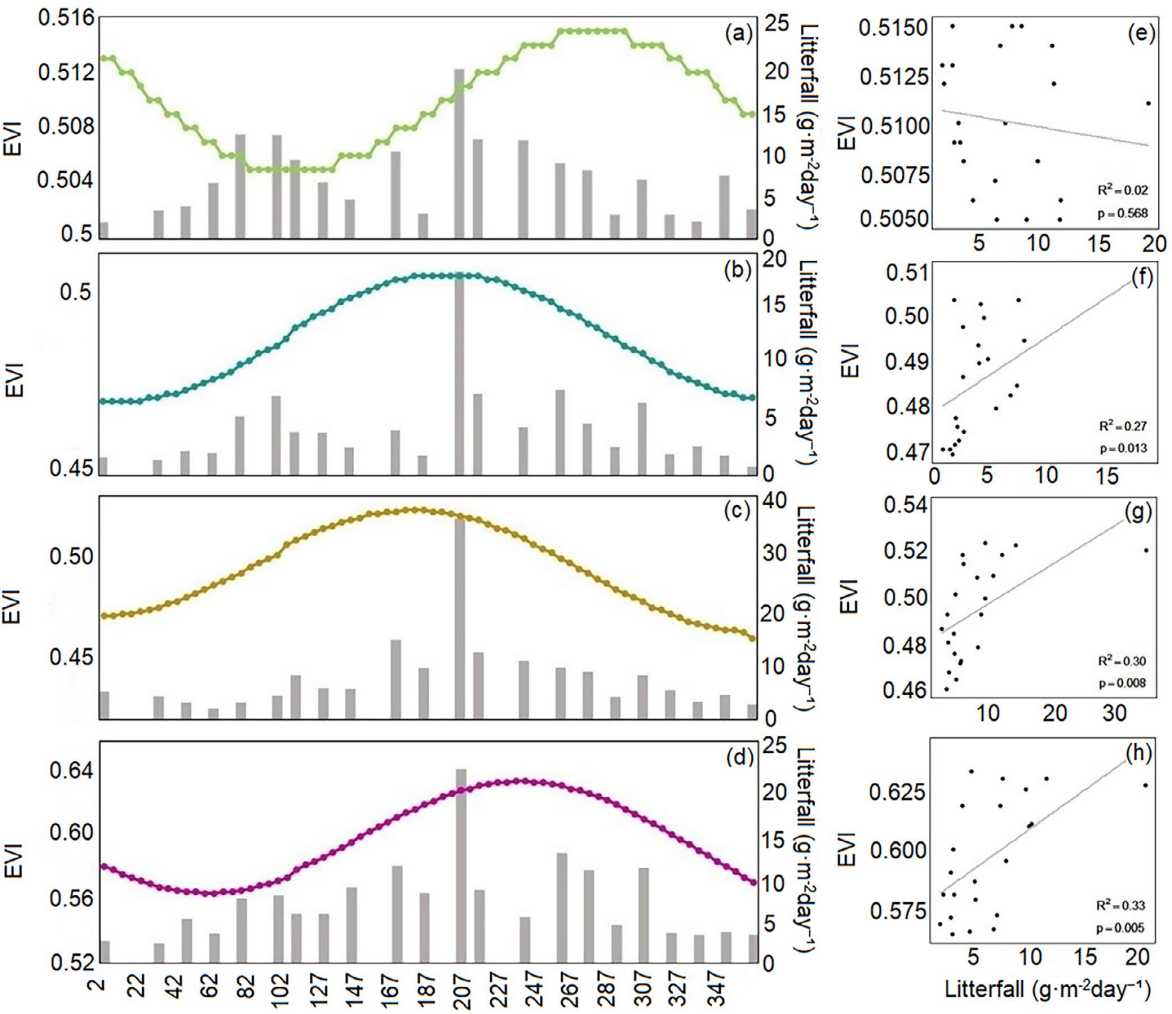
Land surface phenology of four mangrove species at plot scale and the litterfall for the year 2023. Mean values for each species of the three plots are shown in all panels. Scatterplots describing the relationship between EVI values and litterfall for the year 2023 (*n* = 22). *A. corniculatum* (a, e), *A. marina* (b, f), *B. gymnorrhiza* (c, g) and *R. stylosa* (d, h) respectively.

The litterfall dynamics of all four mangrove species reached their highest levels in July (Figure 10a–d). For *A. marina* , *B. gymnorhiza*, and *R. stylosa*, litterfall mass in 2023 was significantly positively correlated with EVI (*p* < 0.05), whereas *A. corniculatum* showed a weak negative correlation (*p* > 0.1).

## 4 Discussion

### 4.1 Mangrove Phenology Derived From Satellite Observations

Satellite remote sensing provides spatially explicit and temporally continuous phenological information, substantially reducing the labor and logistical constraints associated with field‐based monitoring. Previous studies have shown that mangrove phenology exhibits clear seasonality that differs markedly from adjacent terrestrial tropical forests (Songsom et al. 2019). Despite their evergreen nature and distribution in cloudy, tidal coastal zones, mangrove forests still exhibit discernible land surface phenological signals detectable at both regional and plot scales.

Studies conducted in diverse tropical and subtropical regions—including Yucatán Peninsula in Mexico (Pastor‐Guzman et al. 2018), southern Thailand (Songsom et al. 2019), northern Australia (Younes et al. 2020; Younes et al. 2021), and the Gulf of Mexico (Celis‐Hernandez et al. 2022)—have consistently documented strong seasonality in mangroves, although the specific patterns differ regionally. In our study along the northern subtropical coast of China, we observed a clear unimodal phenology curve, consistent with patterns reported in Mexico and Thailand but contrasting with the bimodal signature documented in northern Australia. Two major factors likely explain the interregional differences in mangrove phenology. First, climatic drivers, particularly the timing and intensity of wet and dry seasons, strongly influence canopy productivity and leaf dynamics (Table S4). Second, tidal and geomorphological conditions shape local hydrological regimes, affecting oxygen availability, salinity, and physiological stress responses that modulate phenological timing.

While the general shape of the phenology curve was consistent across scales, the absolute values differed. Regional‐scale EVI was systematically lower than plot‐scale EVI, likely because spatial aggregation smooths fine‐scale canopy heterogeneity and dampens seasonal amplitude (van Bussel et al. 2011). Importantly, the discrepancies between scales cannot be attributed solely to spatial resolution. Additional confounding factors—such as differences in species composition within larger pixels, canopy cover variation, and occasional disturbances (pest outbreaks, storm damage)—may also influence scale‐dependent phenological signals. Overall, these findings demonstrate that optical satellite observations can robustly capture mangrove phenology and reveal meaningful interspecific and spatial variation.

### 4.2 Applicability of Different Remote Sensing Datasets for Mangrove Phenology

Satellite remote sensing has been widely applied to detect vegetation phenological transitions based on temporal trajectories of greenness indices. Freely available archives such as Advanced Very High Resolution Radiometer (AVHRR), MODIS (Moderate Resolution Imaging Spectroradiometer), Landsat, and Sentinel‐2 enable phenology assessments across spatial scales ranging from several kilometers to 10 m. For mangrove ecosystems, MODIS, Landsat, and Sentinel‐2 have been applied to derive mangrove phenology. Mangrove phenology derived from Landsat and Sentinel‐2 sensors is comparable but not from MODIS (Younes et al. 2021). In our study, direct comparison of Landsat 8 and Sentinel‐2 revealed notable and quantifiable differences. Sentinel‐2 exhibited a wider EVI range and stronger short‐term fluctuations, reflecting its higher sensitivity to within‐stand heterogeneity and canopy structural variability. Phenological metrics derived from Sentinel‐2 also showed systematically earlier SOS, later EOS, and longer LOS than Landsat 8, consistent with the expectation that higher temporal resolution enhances the detection of subtle phenological transitions in evergreen systems with prolonged or gradual seasonal cycles. Quantitative statistics further support these differences: the fitted EVI curves showed only a moderate correlation but small discrepancies in absolute values, indicating similar seasonal trajectories but sensor‐specific variation in curve amplitude and detail.

These discrepancies arise not only from spatial resolution but also from differences in spectral response functions, revisit frequency, and noise characteristics, all of which influence phenological signal detection. For local‐ or species‐level phenology, datasets with dense temporal sampling and finer spatial resolution—such as Sentinel‐2—are therefore essential for capturing subtle canopy changes and species‐specific dynamics. Conversely, Landsat remains valuable for regional‐scale assessments, where long‐term consistency and broader spatial coverage are prioritized, even though fine‐grained phenological features may be smoothed or overlooked. Collectively, these interacting factors highlight the need to select data sources that align with the ecological scale and processes of interest and underscore the advantages of combining multiple sensors to mitigate dataset‐specific limitations and improve phenological characterization in mangrove ecosystems.

### 4.3 Species‐Specific Mangrove Phenology

**FIGURE 11:**
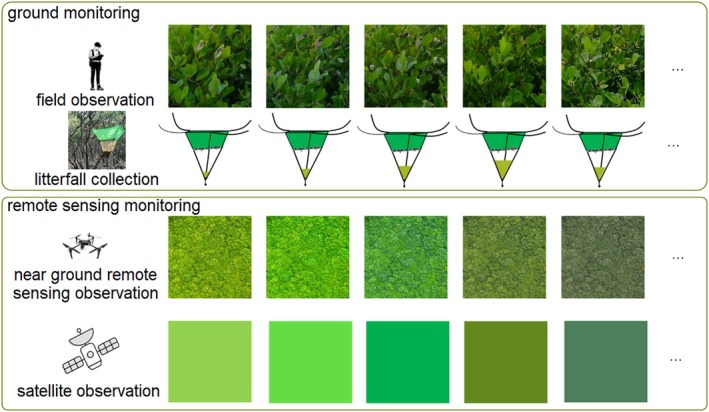
The ground and remote sensing monitoring for mangrove phenology.

Recent advances in remote sensing have demonstrated that phenological signals can capture species‐level differences in mangrove growth dynamics, even within structurally similar evergreen canopies (Small and Sousa 2019; Zhao et al. 2024). In our study, four dominant species exhibited distinct SOS, PGS, and EOS timings, despite all being evergreen. Field observations corroborate these differences: leaf emergence, greenness transitions, flowering, senescence, and litterfall vary substantially across species (Figure 11). For instance, in our study area, the selected species have distinct flowering times: *A. corniculatum* mainly start in January, *A. marina* mainly in May, *B. gymnorrhiza* throughout the year, and *R. stylosa* primarily in the end of August (Table S5). Although optical satellites primarily detect canopy greenness rather than specific reproductive events, the timing of SOS, PGS, and EOS can approximate overall growth patterns, with some expected lag. Because mangroves canopies are dense and evergreen, satellite‐derived phenology typically responds more slowly to subtle changes in leaf age or turnover. Earlier studies reported a lag of 2–3 months between satellite‐based EVI and leaf production measurements (Younes et al. 2020).

Our findings also add nuance to ongoing discussions regarding the universality of mangrove phenological trajectories. Previous studies reported that *A. corniculatum* exhibits a bimodal phenological pattern in northern Australia and the Beibu Gulf of China (Yao et al. 2025), whereas the other common species show unimodal seasonality. In contrast, our study detected a unimodal trajectory for *A. corniculatum*. This divergence may reflect regional climatic constraints, particularly lower winter temperatures that suppress secondary canopy flushing events observed in tropical climates. Such differences underscore the importance of climatic context when interpreting species‐specific phenology from satellite observations.

The relationship between litterfall and satellite‐derived phenology also varied across species. While earlier studies reported negative correlations between litterfall and vegetation indices (Pastor‐Guzman et al. 2018), our results revealed positive correlations for *A. marina* , *B. gymnorhiza*, and *R. stylosa*, and a negative relationship for *A. corniculatum*. These contrasting patterns likely arise from species‐specific differences in canopy architecture, leaf lifespan, and turnover strategies. The seasonal alignment between peak EVI and peak litterfall in our study area—occurring during July and August—highlights the strong coupling between canopy dynamics and regional climate. High temperatures and abundant rainfall during the summer months promote both vigorous canopy growth and intensified leaf turnover. Comparisons with regions such as the Yucatán Peninsula (Pastor‐Guzman et al. 2018) suggest that climatic controls on litterfall seasonality vary substantially among mangrove biogeographic zones, reinforcing the need for region‐specific phenological assessments.

### 4.4 Uncertainties and Limitations

Satellite‐derived mangrove phenology is subject to several uncertainties associated with coastal environments. Although high cloud cover images were excluded and the study area's distribution was meticulously checked using historical high‐resolution images, the daily tidal effects could not be entirely excluded without compromising the high temporal resolution of the time series.

The choice of vegetation index and phenology extraction method also influences results. While numerous VIs (e.g., NDVI, EVI, NDWI) have been used for phenological studies (Wang et al. 2019), we selected EVI for its documented superiority in mangroves (Younes et al. 2021). Because our goal was to compare phenology across scales, datasets, and species, we did not evaluate cross‐VI differences.

Similarly, various methods have been proposed to reconstruct the VI time series for mangroves, including Discrete Fourier Transform (Moody and Johnson 2001; Pastor‐Guzman et al. 2018), Savitzky–Golay filter (Songsom et al. 2019), Generalized Additive Models (Chen et al. 2004; (Younes et al. 2020; Younes et al. 2021), and Harmonic Analysis (Celis‐Hernandez et al. 2022; Roerink et al. 2000). We selected HANTS due to its strong performance in coastal environments and its stability when handling irregular time intervals (Celis‐Hernandez et al. 2022). Additionally, multiple approaches have been proposed to acquire vegetation phenology metrics. Among them, several methods have been used to extract mangrove phenology metrics, including derivative (Dash et al. 2010; Pastor‐Guzman et al. 2018), percentile thresholds (Songsom et al. 2019; Verger et al. 2016) and inflection (Celis‐Hernandez et al. 2022; Jeong et al. 2011). After testing, we adopted the inflection‐point method as the most appropriate for capturing subtle seasonal transitions in evergreen mangrove canopies.

Finally, discrepancies between modeled and observed EVI highlight the need for additional ground validation. Litterfall provided useful reference data, yet only 1 year was available for comparison. More extensive field measurements—particularly leaf area, canopy colorimetry, sap flow, and phenocam imagery—would enhance calibration and improve interpretation of satellite‐observed phenology.

## 5 Conclusions

This study examined the extent to which mangrove phenology can be reliably derived from satellite observations, with a specific focus on three dimensions: spatial scale, satellite dataset characteristics, and species‐level variation. Rather than prescribing a single phenology modeling approach, our aim was to illustrate the complexity of satellite‐based mangrove phenology and provide guidance for future investigations. First, addressing Research Question 1 (scale), we found that regional‐ and plot‐scale analyses yielded broadly consistent and clearly defined phenological patterns. This demonstrates that mangrove phenology can be effectively captured even at coarser spatial resolutions, supporting the feasibility of applying regional‐scale satellite data for long‐term phenological monitoring. Second, in relation to Research Question 2 (dataset differences), our comparison of Landsat 8 and Sentinel‐2 revealed substantial differences in phenology metrics, with Sentinel‐2's higher spatial and temporal resolution enabling more precise detection of seasonal transitions. These results highlight the superior potential of high‐resolution datasets for characterizing mangrove phenology, particularly in heterogeneous or species‐diverse stands. Third, for Research Question 3 (species differences), we showed that species‐specific phenological characteristics can be distinguished using Sentinel‐2 observations at the plot scale. Distinct SOS, EOS, and PGS patterns were evident across the four dominant mangrove species, demonstrating the capability of satellite‐derived indices to capture meaningful interspecific variability. Although satellite‐derived phenology is less temporally precise than field‐based measurements, it nonetheless provides valuable insights into vegetation seasonal dynamics—especially for evergreen, dense‐canopy ecosystems such as mangroves, where ground monitoring is often logistically challenging. Understanding these phenological patterns contributes to improved species‐ and habitat‐level management under changing environmental conditions. Looking forward, we emphasize the importance of integrating ground observations, proximal sensing (e.g., phenocams, UAVs), and multi‐sensor satellite datasets to enhance the accuracy and ecological relevance of mangrove phenology assessments. Such integrative approaches will support more robust monitoring frameworks for coastal ecosystem conservation and climate adaptation.

## Author Contributions

**Yuhang Wang:** conceptualization (equal), formal analysis (equal), funding acquisition (equal), methodology (equal), resources (equal), software (equal), supervision (equal), writing – original draft (equal), writing – review and editing (equal). **Qi Liu:** conceptualization (equal), data curation (equal), investigation (equal). **Yaojun Zhu:** writing – review and editing (equal). **Wanyu Wen:** writing – review and editing (equal). **Si Yang:** writing – review and editing (equal). **Minghao Gong:** writing – review and editing (equal).

## Funding

This work was supported by Fundamental Research Funds of Chinese Academy of Forestry (CAFYBB2020SY045). National Natural Science Foundation of China (42001103).

## Conflicts of Interest

The authors declare no conflicts of interest.

## Supporting information


**Data S1:** ece372788‐sup‐0001‐DataS1.zip.

## Data Availability

The Landsat 8 Level‐2 Surface Reflectance data used in this study are publicly available from the U.S. Geological Survey (USGS) through the Google Earth Engine platform (https://earthengine.google.com/). Sentinel‐2 Level‐2A Surface Reflectance data are provided by the European Space Agency (ESA) under the Copernicus Open Access License and are likewise accessible via Google Earth Engine. The processed remote sensing data products and derived phenology metrics can be made available upon reasonable request from the corresponding author. Litterfall data collected during fieldwork from January 2023 to January 2024 are not publicly archived due to local data use agreements but are available from the corresponding author upon reasonable request.
