# Supplementary material for: Mangrove Phenology From Scale, Data and Species Perspectives
Source: Ecol Evol. 2026 Jan 4;16(1):e72788. doi: 10.1002/ece3.72788 (PMC12765594; doi:10.1002/ece3.72788)
Supplement: Supplementary file 1 — Data S1: ece372788‐sup‐0001‐DataS1.zip. [file ECE3-16-e72788-s001.zip › Suppoorting information.docx]

Supporting information

Table. S1 The geospatial coordinates of field plots, All photos were taken by the authors (Yuhang Wang) in the Gaoqiao Mangrove Reserve during field campaigns in 2023. Images used with permission from the photographers.

| Site species | plot type | longitude | latitude | photographic documentation of sampling sites |
| --- | --- | --- | --- | --- |
| *A. corniculatum* | *A. corniculatum* plot1 | 109.767278 | 21.551307 | 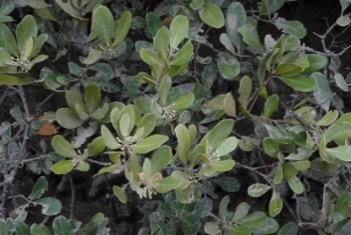 |
|  | *A. corniculatum* plot2 | 109.767337 | 21.553009 |  |
|  | *A. corniculatum* plot3 | 109.768164 | 21.555009 |  |
| *A. marina* | *A. marina* plot1 | 109.781839 | 21.532297 | 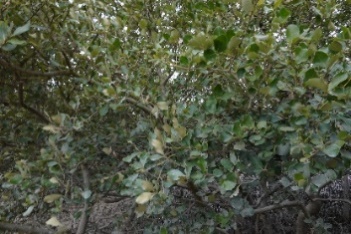 |
|  | *A. marina* plot2 | 109.784258 | 21.531895 |  |
|  | *A. marina* plot3 | 109.784846 | 21.531786 |  |
| *B. gymnorhiza* | *B. gymnorhiza* plot1 | 109.783986 | 21.529072 | 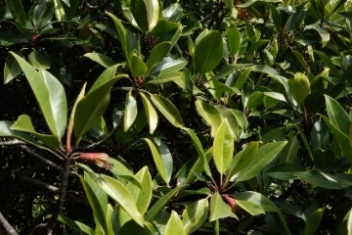 |
|  | *B. gymnorhiza* plot2 | 109.786163 | 21.528408 |  |
|  | *B. gymnorhiza* plot3 | 109.784588 | 21.528468 |  |
| *R. stylosa* | *R. stylosa* plot1 | 109.767799 | 21.548536 | 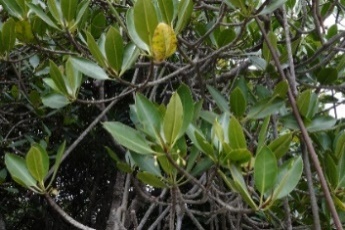 |
|  | *R. stylosa* plot2 | 109.768332 | 21.548208 |  |
|  | *R. stylosa* plot3 | 109.767864 | 21.549100 |  |


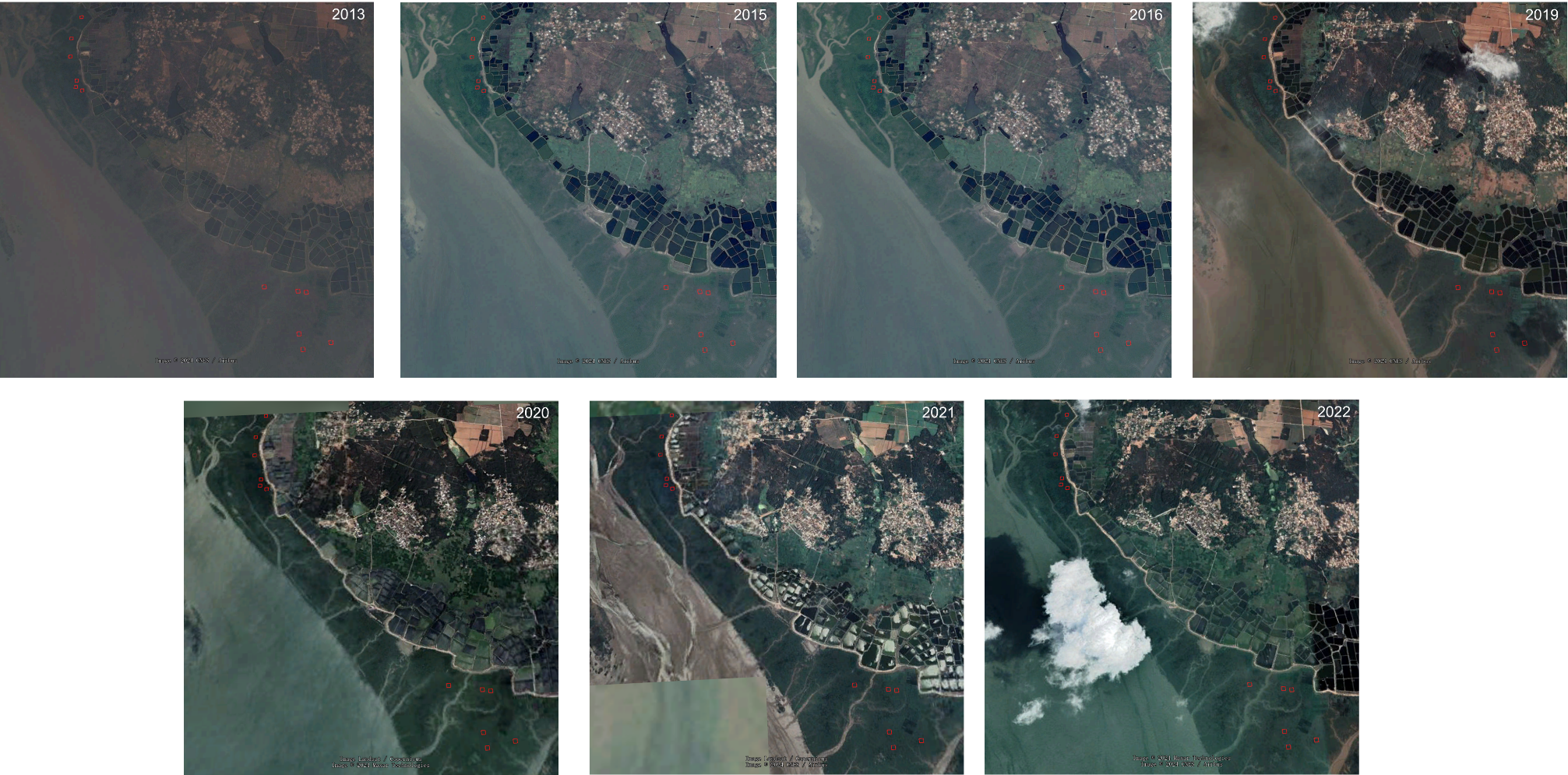


Fig. S1 The land cover for the selected plots from 2014 to 2023 using Google Earth historical images. The red squares are the selected plots within the study area.


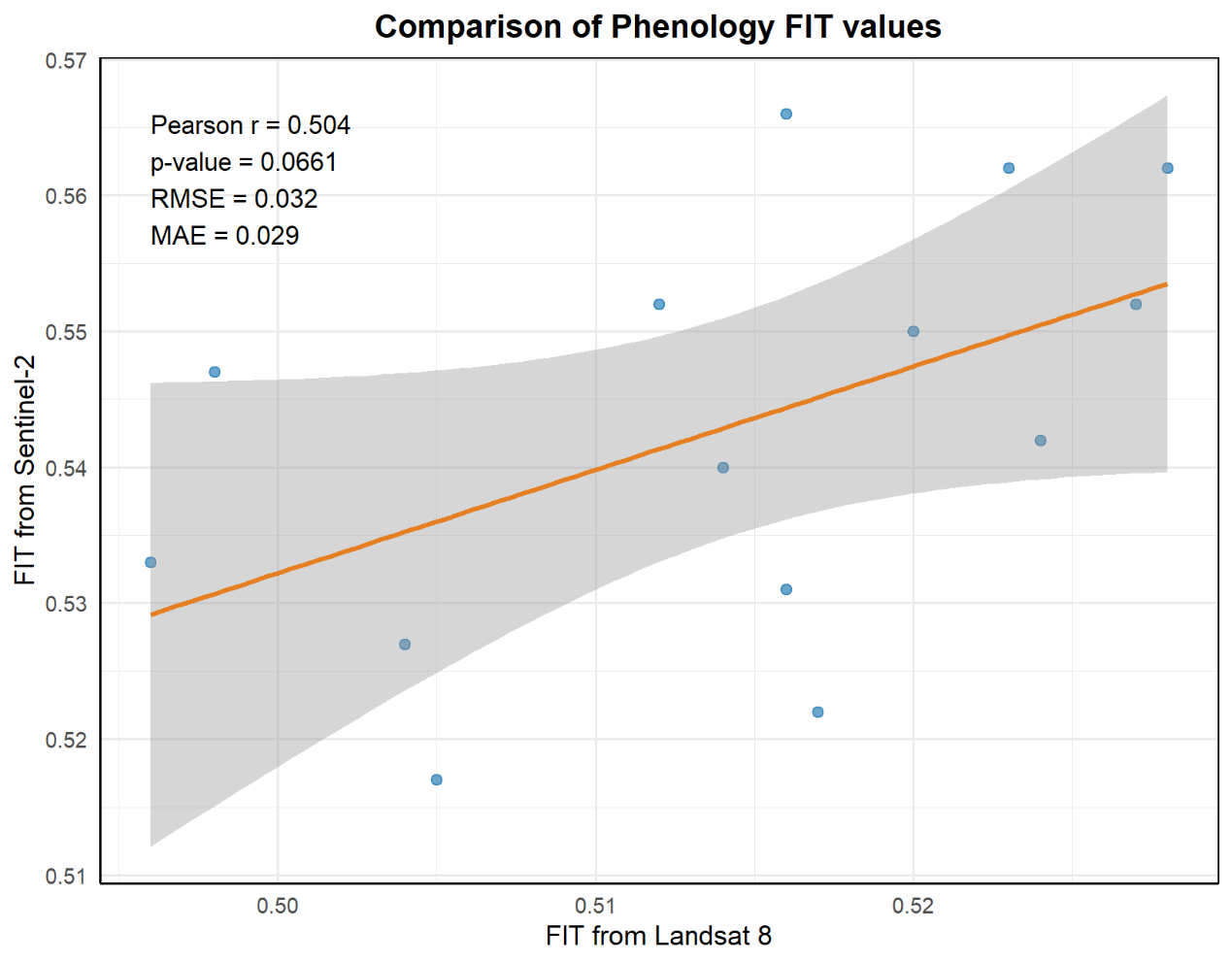


Fig S2. Scatterplot comparing fitted EVI values (FIT) derived from Landsat 8 and Sentinel-2 at the plot scale. Each point represents a paired phenology estimate obtained from temporally matched observations. The solid orange line indicates the least-squares regression fit, with the shaded area denoting the 95% confidence interval. Statistical metrics are shown in the upper-left corner, including Pearson correlation coefficient (r), significance level (p-value), root-mean-square error (RMSE), and mean absolute error (MAE). Together, these results indicate moderate correspondence between Landsat- and Sentinel-derived phenology trajectories, with small absolute deviations in fitted EVI values.

Table S2 Compilation of mean SOS, EOS, LOG and POG each year for question 1

| year | scale | SOS | EOS | LOG | POG |
| --- | --- | --- | --- | --- | --- |
| 2014 | plot | 46 | 344 | 220 | 220 |
|  | region | 43 | 345 | 220 | 220 |
| 2015 | plot | 39 | 337 | 220 | 220 |
|  | region | 39 | 343 | 220 | 220 |
| 2016 | plot | 56 | 346 | 219 | 219 |
|  | region | 52 | 349 | 221 | 221 |
| 2017 | plot | 44 | 346 | 218 | 218 |
|  | region | 41 | 349 | 219 | 219 |
| 2018 | plot | 47 | 346 | 218 | 218 |
|  | region | 46 | 352 | 220 | 220 |
| 2019 | plot | 49 | 352 | 220 | 220 |
|  | region | 43 | 355 | 220 | 220 |
| 2020 | plot | 41 | 328 | 219 | 219 |
|  | region | 40 | 329 | 220 | 220 |
| 2021 | plot | 37 | 343 | 218 | 218 |
|  | region | 39 | 346 | 219 | 219 |
| 2022 | plot | 38 | 344 | 218 | 218 |
|  | region | 40 | 349 | 219 | 219 |
| 2023 | plot | 38 | 339 | 221 | 221 |
|  | region | 40 | 339 | 220 | 220 |

Table S3 Compilation of mean SOS, EOS, LOG and POG each year for question 2

| year | scale | SOS | EOS | LOG | POG |
| --- | --- | --- | --- | --- | --- |
| 2019 | Landsat 8 | 92 | 364 | 272 | 252 |
|  | Sentinel-2 | 53 | 363 | 310 | 213 |
| 2020 | Landsat 8 | 111 | 335 | 224 | 255 |
|  | Sentinel-2 | 53 | 363 | 310 | 213 |
| 2021 | Landsat 8 | 97 | 353 | 256 | 257 |
|  | Sentinel-2 | 52 | 362 | 310 | 212 |
| 2022 | Landsat 8 | 84 | 356 | 272 | 260 |
|  | Sentinel-2 | 52 | 362 | 310 | 212 |
| 2023 | Landsat 8 | 71 | 359 | 288 | 263 |
|  | Sentinel-2 | 52 | 362 | 310 | 207 |

Table S4 Comparison for the current mangrove phenology studies based on satellite observation

| Study area | | Study area climate | | Dataset | | Phenology characteristics | | Reference | |
| --- | --- | --- | --- | --- | --- | --- | --- | --- | --- |
| Yucatan Peninsula, south east Mexico | | a dry season from March to May, a rainy season from June to October and a cold season from November to February | | MODIS | | SOS ranged between day of the year (DOY) 144 (late dry season) and DOY 220 (rainy season) while the EOS occurred between DOY 104 (mid-dry season) to DOY 160 (early rainy season). The length of the growing season ranged between 228 and 264 days | | Pastor-Guzman et al., 2018 | |
| southern coast of Thailand (five sites: Ranong province, Phang-nga province, Krabi province, Trang province, and Nakhon Si Thammarat province.) | wet and dry seasons: (dry season months: Ranong and Trang: Feb to May, Phang-nga, Krabi and Nakhon Si Thammaratang: Jan to Apr) | | MODIS | | starting in April to June, peaking in August to October and ending in January to February of the following year | | Songsom et al., 2019 | |  |
| Northern Australia | | a tropical climate with high humidity and two seasons, the wet (October to April) and dry season (May to September). During the dry season nearly every day is warm and sunny, and afternoon humidity averages around 30%. | | Landsat | | a bimodal seasonality: start of season and peak growing season dates: the former occurs between September and October and the latter May and July. | | Younes et al., 2020 | |
| Darwin Harbour (Northern Territory, Australia) | | a tropical climate with high humidity and two seasons, the wet (October to April) and dry season (May to September). During the dry season nearly every day is warm and sunny, and afternoon humidity averages around 31%. | | MODIS, landsat, Sentinel-2 | | a bimodal seasonality: start of season and peak growing season dates: the former occurs between September and October and the latter May and July. | | Younes et al., 2021 | |
| Isla del Carmen, Mexico | | The weather is generally warm with summer rainfall. The average annual temperature and rainfall are 26.7 °C and 1900 mm, ranging between 23.9 °C and 28.8 °C, and 1174 mm and 3139mm, respectively. Seasonally, three different regimes are recognizable, the dry season (Feb–May), rainy season (Jun–Sep), and stormy season (Oct–Jan). | | Sentinel-2 | | starting in May, peaking in January to February and ending in April to May of the following year | | Celis-Hernandez et al., 2022 | |

Table. S5 The flowering for the four selected species within the study area

| Species | Bud stage | flowering stage | full flowering stage |
| --- | --- | --- | --- |
| *A. corniculatum* | January-March | January-March | March-April |
| *A. marina* | March-May | April-May | April-May |
| *B. gymnorrhiza* | all the year | | |
| *R. stylosa* | April-September | August-October | October-December |

the data was from the ground observation from 2019 to 2023
